# Population dataset for 23 Y-STR in the Merkit clan form Kazakh population

**DOI:** 10.1016/j.dib.2024.110160

**Published:** 2024-02-09

**Authors:** Bekzhan Faizov, Alizhan Bukayev, Zhaxylyk Sabitov, Maxat Zhabagin

**Affiliations:** a National Center for Biotechnology, Astana 010000, Kazakhstan; b Research Institute for Jochi Ulus Studies, Astana 010000, Kazakhstan

**Keywords:** Population genetics, Haplotype, STR, PowerPlex Y-23, STRAF, Merkit clan, Kazakh population

## Abstract

This study presents a comprehensive analysis of 23 Y-STR data for the Merkit clan, a subgroup within the Kerey tribe of the Kazakh people. A total of 64 complete haplotypes were generated using the PowerPlex Y23 System. The data obtained using 23 Y-STR markers has been submitted to the Y Chromosome Haplotype Reference Database (YHRD) at yhrd.org, which will significantly enhance the forensic database for the Kazakh population in Kazakhstan. The research focuses on the distribution of haplotypes within the clan and their genealogical lines, which were visualized using a Median-joining network and Multidimensional scaling plot. The study identifies four distinct haplogroup clusters, revealing important insights into the genetic makeup and historical lineage of the Merkits. This dataset not only enriches our understanding of Kazakh genetic structure but also holds significant value for anthropological and population genetic research, as well as for forensic genetics. This work bridges a notable gap in genetic research on the Merkit clan, contributing to a deeper understanding of Central Asian nomadic tribes.

Specifications Table

**Table:** 

Subject	Genetics
Specific subject area	Forensic Genetics, DNA Profiling, Population Genetics
Data format	Raw and Analyzed
Type of data	Supplementary tables (5), table (1) and figures (3)
Data collection	Saliva samples were collected using the Oragene DNA (OG-500) Self-Collection Kit (DNA Genotek, Canada). Samples were obtained from healthy Kazakh male volunteers, each confirmed not to be related to any other participant for at least three generations and knowledgeable about their clan affiliation. DNA isolation from the saliva was performed using the prepIT-L2P kit (DNA Genotek, Canada). The DNA concentration and quality were determined using a Qubit 2.0 Fluorometer and NanoDrop One Spectrophotometry, respectively (both from Thermo Fisher Scientific, USA). PCR amplification was performed using the PowerPlex Y23 System (Promega, USA) in a reaction mixture on a SimpliAmp Thermal Cycler (Thermo Fisher Scientific, USA). Electrophoresis for separating PCR products was conducted using WEN Internal Lane Standard 500 (Promega, USA) in Hi-Di Formamide on an 8 capillary Applied Biosystems 3500 genetic analyzer with POP-4 polymer, Cathode and Anode buffers (Thermo Fisher Scientific, USA). STR allele calls in electropherograms were determined using GeneMapper IDx v.1.6 software. Further data analysis was conducted using Microsoft Office Excel, Arlequin program, STRAF Software 2.1.5, and Haplomatch tool.
Data source location	Laboratory of Human Genetics, National Center for Biotechnology, Astana, 010000, Kazakhstan
Data accessibility	Repository name: YHRD - Y-chromosome haplotype research database Data identification number: YA006027 Direct URL to data: https://yhrd.org/details/contribution/6027 Instructions for accessing these data: All data are available with the article as supplementary files.

## 1 Value of the Data

• The data of 23 Y-STR profiles from the Merkit clan reveals their paternal signature within the Kazakh population, illuminating sub-population differentiation and enriching our understanding of Kazakh genetic structure.
• Contributing Merkit clan Y-STR profiles to the Y-chromosomal Haplotype Reference Database enhances the accuracy and discriminatory power of DNA evidence in kinship analysis, criminal investigations, and missing persons’ cases for forensic laboratories worldwide.
• This dataset is a valuable resource for anthropological, population genetic, and evolutionary research, providing insights into the unique genetic makeup of the Kazakh population and illuminating the genetic connections between Kazakhs and other global populations.
• The dataset, encompassing 23 Y-STR profiles from the Merkit clan, significantly contributes to the advancement of genetic genealogy, providing interdisciplinary insights to elucidate the demographic and historical events.

## 2 Background

Y-chromosomal short tandem repeats (Y-STRs) are integral tools in forensic science, providing critical insights for criminal investigations and legal proceedings. Additionally, these genetic markers are extensively utilized in genealogical research, anthropology, and population genetics, aiding in the tracing of paternal lineages and understanding population structures and migrations. In Kazakh society, the affiliation of each male individual to a particular clan is a cultural practice that has been preserved. Each clan is characterized by its own distinct history, origin, and genealogy, which is traditionally traced back to a shared common ancestor. However, these ethnographic records often vary. Today, advancements in genetic research, particularly the analysis of the Y-chromosome, provide a scientific means to verify and augment these ethnographic data. Consequently, there is a pressing need to delineate the genetic diversity of Y-chromosomes among Kazakh clans. In this study, we have presented a dataset of haplotypes based on 23 Y-STR markers specifically for the Merkit clan. These datasets expand the Y-chromosome haplotype research database, providing valuable resources for both forensic genetics and genetic genealogy.

## 3 Data Description

In this brief article, we present 23 Y-STR data for the Merkit clan, a subgroup within the Kerey tribe of the Kazakh people. A total of 64 complete haplotypes were generated using the PowerPlex Y23 System (Promega, USA). The Merkits have been an integral part of the broader narrative of Mongolia and Central Asian history [1]. Their lineage, often intertwined with the vast and complex history of nomadic tribes in the region, offers a rich context for genetic studies. Despite their historical significance, there has been a notable gap in genetic research specifically focused on the Merkit clan. This study aims to bridge this gap by providing paternal genetic data, which is crucial for understanding not only the genetic makeup of the Merkits, but also their relationship and distinction within the wider Kazakh population. The lack of prior genetic studies on the Merkit clan underscores the importance of this research, as it contributes to a more comprehensive understanding of the genetic diversity and historical migrations of Central Asian nomadic tribes. Furthermore, this genetic data holds immense value for forensic genetics. The unique Y-STR profiles of the Merkit clan can significantly enhance the accuracy and reliability of DNA-based analyses in forensic science.

**Fig. 1:**
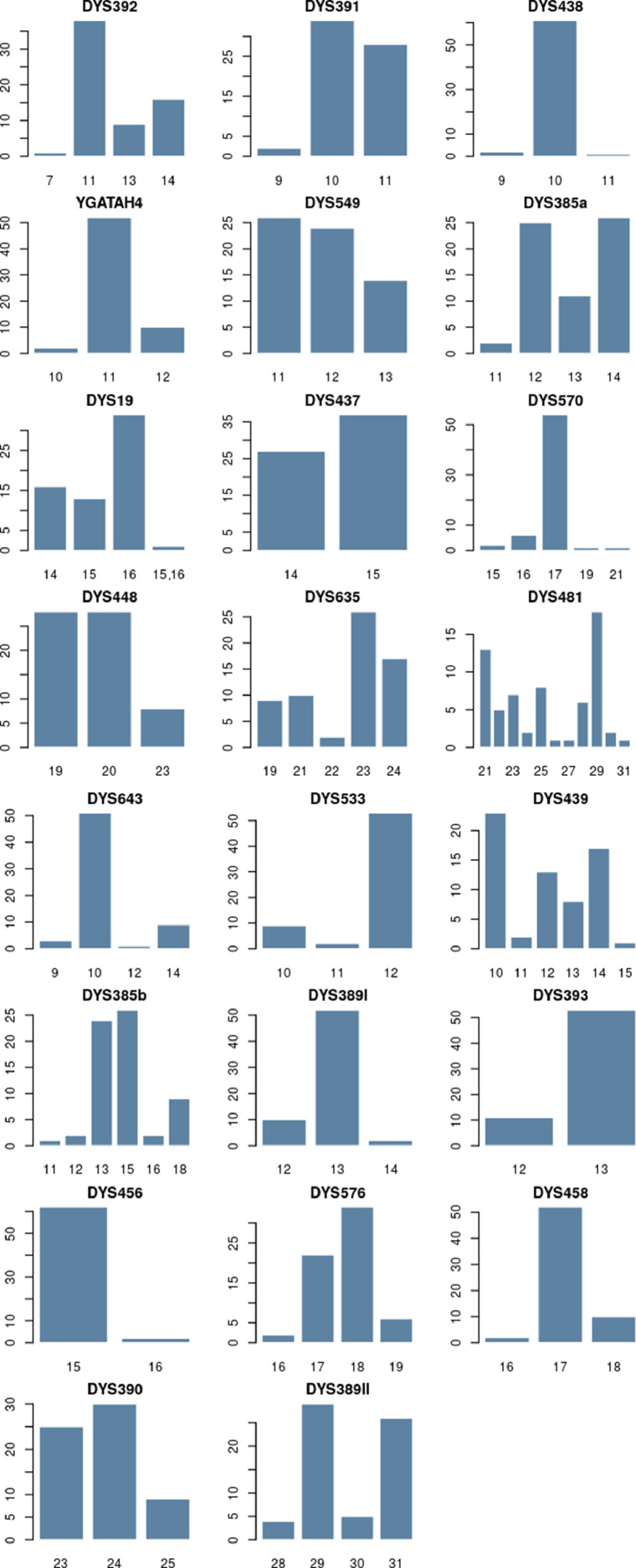
Distribution of allelic frequencies per locus on 23 Y-STR loci in Merkit clan form Kazakh population using STRAF software.

The distribution of 23 Y-STR haplotypes within the Merkit clan from Kazakhstan, involving 64 individuals, is detailed in Supplementary Table 1. Analysis of haplotype frequencies identified 33 distinct haplotypes, as shown in Supplementary Table 2. Among these, 7 haplotypes were shared by pairs of individuals, and one haplotype was shared by groups of three, four, and six individuals. The two most prevalent haplotypes were each shared among eight individuals. Forensic parameters that describe the Merkit clan were computed, including Discrimination Capacity (DC = 52%), Haplotype Match Probability (HPM = 0.06), and Haplotype Diversity (HD = 0.96). Allele frequencies and forensic parameter values for 23 individual Y-STR loci in the Merkit clan are presented in Fig. 1 and Supplementary Tables 3 and 4. The Y-STR profiling revealed 80 alleles at single-copy loci, with frequencies ranging from 0.02 to 0.95. The minimum number of allelic variants (n = 2) was observed at the DYS393, DYS437, and DYS456 loci. The DYS481 locus exhibited the highest number of allelic variants (n = 11). Gene diversity (GD) among the Merkits varied from 0.09 at DYS438 to 0.85 at DYS481. Contrary to the standard loci, the average gene diversity of the six rapidly mutating single-copy loci (GD = 0.51) was better compared to the fifteen standard single-copy loci (GD = 0.46). The lowest GD values in rapidly mutating loci were observed at DYS533 (GD = 0.30) and DYS570 (GD = 0.28). Among the standard loci, the highest GD values were recorded at DYS439 (GD = 0.75) and DYS635 (GD = 0.73), which are on par with those observed in rapidly mutating loci. The frequency distribution of specific haplotypes and forensic parameters for the DYS385a/b locus in the Merkits are detailed in Supplementary Table 4, with the locus showing 7 haplotype combinations from 7 distinct alleles. The gene diversity of the DYS385a/b locus is 0.68. One copy number variation was identified in the Merkit clan at the DYS19 locus (alleles 15, 16).

**Table 1:** Indicators of similarity between the founder haplotypes of the Merkits' haplogroup clusters and those of the surrounding Kazakh tribes were calculated using the Haplomatch program.

Hapotype	Haplogroup Cluster	Match Tribe	Sample size	exact matches	one-step neighbors	two-step neighbors	three-step neighbors
HP1	I2a1	Zhalayir	93	0	0	0	6
HP2	N1a2	Kerei	141	3	1	0	0
HP4	O2a2	Naiman	161	17	21	16	8
HP7	C2a1a3	Kerei	101	56	17	2	2

**Fig. 2:**
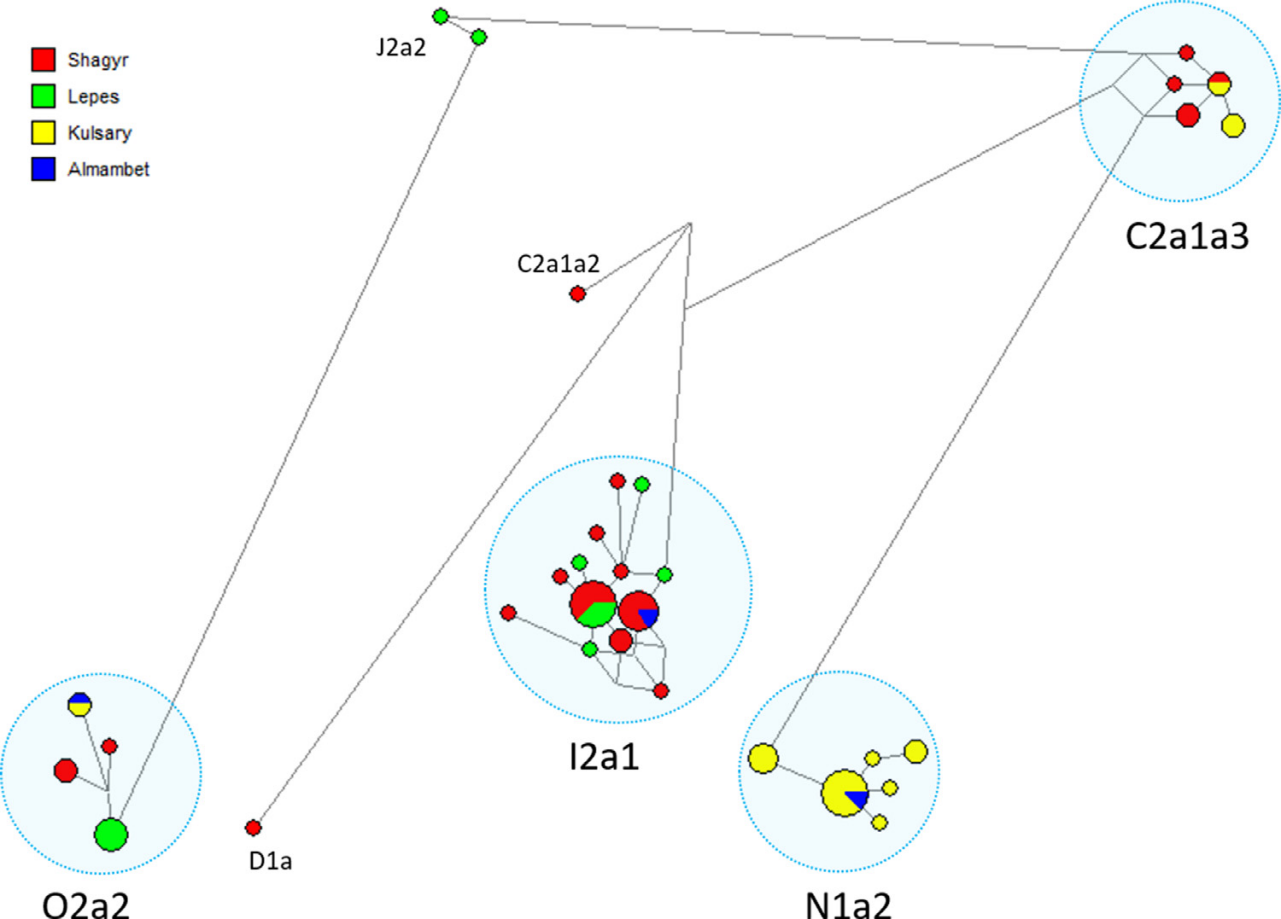
Median-joining network for the haplotypes of 64 Merkit clan members from the Kazakh population was constructed from data on 21 Y-STRs (DYS385a/b loci were excluded). The colors represent the genealogical lineages of the Merkit clan. Circles represent haplotypes, with the area proportional to the sample size, and lines between them proportional to the number of mutational steps. Haplotype clusters are delineated with a dotted circle and annotated according to haplogroups.

**Fig. 3:**
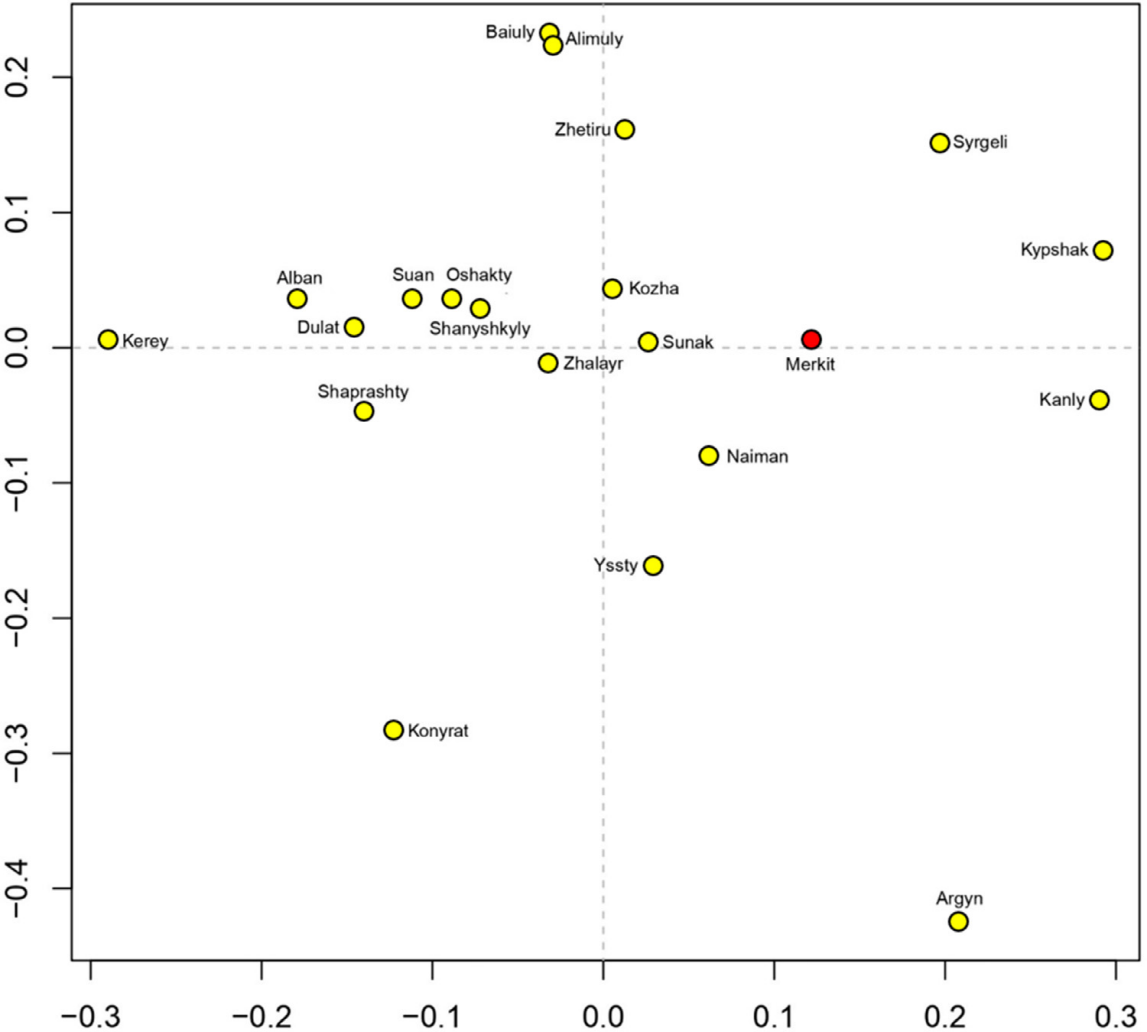
Multidimensional scaling plot based on pairwise genetic distance (RST) between Kazakh clans on 17 Y-STRs.

The distribution of haplotypic diversity within four genealogical lines of the Merkit clan (Almambet, Kulsary, Lepes, Shagyr) is visualized on a Median-joining network for 21 Y-STRs, as presented in Fig. 2. Four haplogroup clusters represent the Merkit haplotypes: C2a1a3-F1918, I2a1-P37.2, N1a2-L666, O2a2-P201. A search for founder haplotypes in the Kazakh haplotype database reveals matches within three mutational steps in other Kazakh tribes (Supplementary Table 2). This indicates genealogical-historical connections between them. In the genetic space, depicted in Fig. 3, the Merkits occupy a distinct position, clearly not forming a genetic subgroup within any tribe.Supplementary Table 5 presents pairwise genetic distances (RST) between Kazakh clans based on 17 Y-STRs. The closest genetic distance is from the Sunak clan (d= 0.1), while the most distinct genetic distances are from the Argyn (d=0.51), Kerey (d=0.45), and Konyrat tribes (RST=0.40).

## 4 Experimental Design, Materials and Methods

### 4.1 Sample Collection

To conduct the research, 64 saliva samples were collected from healthy Kazakh male volunteers. The collection of samples was conducted in accordance with the criteria of the population-genetic biobank [2]. Each volunteer provided informed consent by signing a consent form and answered questions regarding their ancestors' origins to participate in the study. The volunteers, each with knowledge of their clan and genealogical lineage, were confirmed not to be related to each other for at least three paternal generations. Saliva samples were collected using an Oragene DNA (OG-500) Self-Collection Kit (DNA Genotek, Canada) according to the manufacturer's protocol.

### 4.2 DNA Isolation, Amplification and STR Genotyping

DNA was isolated from saliva samples using the prepIT-L2P kit (DNA Genotek, Canada). The DNA concentration was subsequently determined using a Qubit 2.0 Fluorometer (Thermo Fisher Scientific, USA), and DNA quality was assessed via NanoDrop One Spectrophotometry (Thermo Fisher Scientific, USA). PCR (Polymerase Chain Reaction) amplification was performed in a reaction mixture utilizing the PowerPlex Y23 System (Promega, USA) on a SimpliAmp Thermal Cycler (Thermo Fisher Scientific, USA). The PCR products were separated by electrophoresis with WEN Internal Lane Standard 500 (Promega, USA) in Hi-Di Formamide (Thermo Fisher Scientific, USA) using an 8 capillary Applied Biosystems 3500 genetic analyzer with POP-4 polymer, and both Cathode and Anode buffers (Thermo Fisher Scientific, USA). Control DNA 007 (Thermo Fisher Scientific, USA) served as a positive control, while ddH2O was used as a negative control for each genotyping batch. The PowerPlexY23 System (Promega, USA) encompasses 17 conventional Y-STR markers (DYS19, DYS385 a/b, DYS389I/II, DYS390, DYS391, DYS392, DYS393, DYS437, DYS438, DYS439, DYS448, DYS456, DYS458, DYS635, Y-GATA-H4) along with 6 loci known for their high mutation rates (DYS481, DYS533, DYS549, DYS570, DYS576, DYS643). Concurrently with each sample batch, negative and positive controls were run. Samples exhibiting non-standard patterns, off-ladder, and microvariant alleles were repeated. Our laboratories have successfully completed the YHRD Quality Control Test (YC000343) for contributing haplotype data. Following the guidelines for population genetic data [3], the haplotypes were submitted to the YHRD (http://www.yhrd.org) under accession number YA006027. The population genetic data is included in Supplementary Table 1.

### 4.3 Data Analysis

STR allele calls were determined from electropherograms using GeneMapper IDx v.1.6 software. Haplotype frequencies were calculated using the Arlequin program ver 3.5 [4]. We calculated the number of distinct haplotypes, the frequency of unique haplotypes, discrimination capacity, haplotype match probability, and haplotype diversity through direct counting by Microsoft Office Excel. Haplotype diversities were computed using the formula HD = n*(1 − ∑p_i_˄2)/(n − 1), where n represents the sample size and pi the frequency of the i-th haplotype [5]. Haplotype match probability (HMP) was determined using the sum of squared observed haplotype frequencies. Discrimination capacity (DC) was calculated as the ratio of the total distinct haplotypes to the number of haplotypes. Forensic parameters, such as Random Match Probability (RM), Power of Discrimination (PD), Gene Diversity (GD), Polymorphism Information Content (PIC), Power of Exclusion (PE), Typical Paternity Index (TPI), and frequency for each locus, were calculated using the STRAF 2.1.5 [6]. The YHRD website's 'AMOVA and MDS' online tool (http://www.yhrd.org) facilitated the computation of pairwise genetic distances (RST) and multidimensional scaling (MDS). Affiliation of haplotypes to haplogroups was assessed using Nevgen Y-DNA haplogroup predictor (https://www.nevgen.org/) and by comparing haplotypes from published data. The analysis incorporated an extensive dataset, which included 3306 haplotypes for minimum 17 Y-STRs from existing studies on population samples of Kazakhs [7], [8], [9], [10], [11], [12], [13], [14], [15], [16], [17]. In the search for similar haplotypes, the study employed the Haplomatch tool [18] to analyze Y-chromosome STR haplotypes. Median-joining networks were generated using NETWORK v5.0.1.0 and NETWORK Publisher v2.1.2.5 [19]. The DYS385a/b loci were excluded from network construction due to the inability to associate particular alleles with specific copies.

## Limitations

Not applicable.

## Ethics Statement

This research was approved by the Ethics Committee of the National Centre for Biotechnology (protocol No. 5 of 16 October 2020) and was performed following the standards of the Declaration of Helsinki 1964. All participants in this study provided their written informed consent prior to sample collection.

## CRediT authorship contribution statement

**Bekzhan Faizov:** Investigation, Writing – original draft, Visualization. **Alizhan Bukayev:** Investigation, Validation, Project administration. **Zhaxylyk Sabitov:** Conceptualization, Resources, Data curation, Funding acquisition. **Maxat Zhabagin:** Methodology, Software, Formal analysis, Writing – review & editing, Supervision.

## Data Availability

Population dataset for 23 Y-STR in the Merkit clan form Kazakh population (Original data) (Y-chromosome haplotype research database).

## Appendix Supplementary materials

Image, application 1
